# Diabetic Retinopathy Network for the Commonwealth

**Published:** 2015

The new Diabetic Retinopathy Network (DR-NET) links 15 hospitals in 10 low- and middle-income countries to share experiences and support each other to identify and treat more patients with diabetic retinopathy. The need is great: in the catchment areas served by the 15 institutions, nearly 400,000 people are estimated to have diabetic retinopathy (DR), but fewer than 10,000 are currently identified and receiving the appropriate treatment. By identifying patients with diabetic retinopathy early, more people will have their sight saved and the number of people going blind unnecessarily across the Commonwealth will be reduced.

The DR-NET was established in 2014 with a grant from the Queen Elizabeth Diamond Jubilee Trust to the Commonwealth Eye Health Consortium, which is based at the International Centre for Eye Health (ICEH) and works with multiple partners internationally. The project was initiated by the VISION 2020 LINKS Programme, which had been requested by partners to develop DR as a priority area for capacity development and shared learning (**http://iceh.lshtm.ac.uk/vision-2020-links-programme/**). The DR-NET benefits from established VISION 2020 LINKS partnerships between eye units in the UK and Africa plus some from other continents. LINKS in countries outside the Commonwealth also joined the DR-NET to share learning.

The DR-NET was launched with a workshop in November 2014, attended by more than 70 participants. Representatives from each LINK included an ophthalmologist, a diabetologist, a Ministry of Health (MoH) representative and an ophthalmic nurse or DR screener. This representation promoted coordinated planning and ownership of the project by key healthcare workers and the MoH in each country.

Strengths and weaknesses of current service provisions were discussed and each of the participating LINK teams developed a two-year DR detection and treatment plan for their district or country. The teams focussed their discussions on three key questions of planning: ‘Where are we now? Where do we want to be? How do we get there?’

**Figure:**
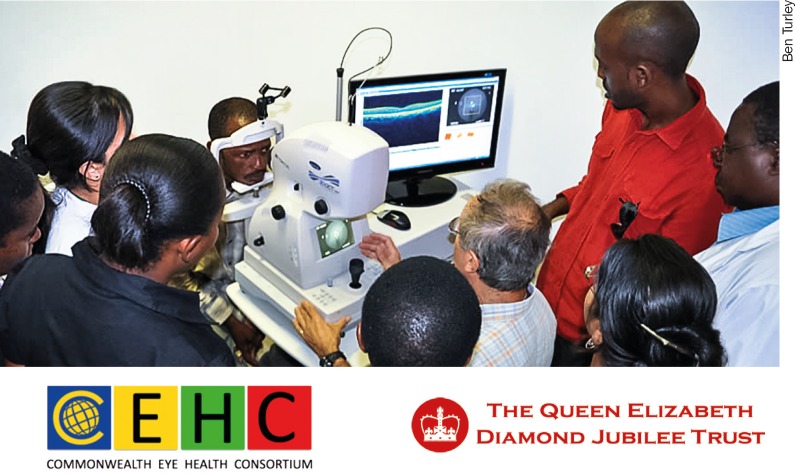
St Thomas' photographer Don Nelson demonstrates OCT to Muhimbili Eye Department

Advocacy with the Ministry of Health and non-governmental agencies was identified as a priority in all the action plans, as financial and political support is needed so that projects can be implemented successfully.

In total, the participating LINKS partners serve approximately 3.8 million people with diabetes, of whom an estimated 10% have visually threatening diabetic retinopathy (VTDR) requiring treatment. Currently less than 2.5% of the people suspected of having VTDR are identified and receiving treatment.

During the workshop, a commitment was made by all the LINKS teams to increase treatment of DR by at least one patient per week. Over the course of the five-year project, the teams will treat at least 3,750 more patients. Assuming an average often years life expectancy at the time of treatment, it is estimated that the DR-NET will prevent a minimum of 37,500 years of blindness.

A key aspect of the project is the development of an ongoing network between the partners for sharing experiences and supporting each other. A virtual networking platform has been established (**https://sites.google.com/site/drnetcomm/**) through which each partner is sharing data on the number of patients screened and treated each month, and also tools that can be used for training, data collection etc.

Over the five years of the project, each of the DR-NET LINKS will work with the MoH in their country to develop a framework for DR services. There will be regular training visits between partners to build capacity for DR screening and treatment services. To ensure sustainability the DR-NET is promoting the integration of services into the general health system with a strong focus on training and capacity-building.

Since the workshop, participants have made remarkable progress on the development of national plans for DR services. For example, a national framework for DR services in Botswana has been developed which also includes the use of the Portable Eye Examination Kit (Peek) in outreach screening.

Although the DR-NET is a time-limited five-year project (until 2019) it is envisaged that the emphasis on building capacity (training, equipment, tools and systems) to identify and treat patients with diabetic retinopathy will result in a lasting legacy to reduce blindness from diabetes in low- and middle-income settings of the Commonwealth.

**Peek Vision**

Peek, the Portable Eye Examination Kit, is a set of diagnostic tools that allows eye care workers to use a smartphone to screen eye patients. It makes use of ‘cloud’-based systems to enable data sharing, referral and follow-up of patients.

Peek Retina is an adapter placed over the camera of a smartphone that enables retinal imaging. It has been trialled in Kenya as a prototype, showing it to be comparable to a desktop camera for optic nerve imaging. Further studies are underway to validate its use for diabetic retinopathy and malaria retinopathy.

**Figure:**
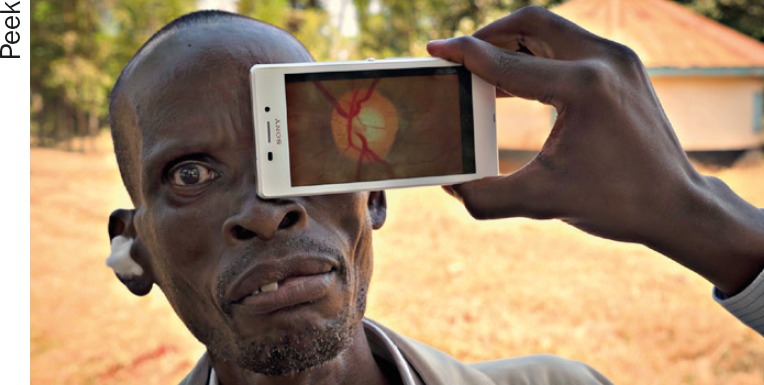
A Peek health care worker screening in the community. KENYA

A diabetic retinopathy screening system including Peek Acuity and Peek Retina is currently being built and trialled in Tanzania. The team hopes to make this widely available once it is fully functional and the programme evaluation is complete.

Peek apps will be free to download from the Google Play store once ready for release. Peek Retina is anticipated to be complete and ready for shipping in 2016. To keep updated on our research, release dates and news, please sign up to our newsletter at **www.peekvision.org**

